# Impact of Household Cooking Techniques on African Nightshade and Chinese Cabbage on Phenolic Compounds, Antinutrients, *in vitro* Antioxidant, and β-Glucosidase Activity

**DOI:** 10.3389/fnut.2020.580550

**Published:** 2020-12-21

**Authors:** Millicent G. Managa, Jerry Shai, Anh Dao Thi Phan, Yasmina Sultanbawa, Dharini Sivakumar

**Affiliations:** ^1^Phytochemical Food Network Research Group, Department of Crop Sciences, Tshwane University of Technology, Pretoria, South Africa; ^2^Department of Biomedical Sciences, Tshwane University of Technology, Pretoria, South Africa; ^3^Agricultural Research Council Industrial Transformation Training Centre for Uniquely Australian Foods, Queensland Alliance for Agriculture and Food Innovation, The University of Queensland, Brisbane, QLD, Australia

**Keywords:** indigenous leafy vegetables, oxalates, β-glucosidase activity, kaempferol derivatives, chlorogenic acid, FRAP activity

## Abstract

Different household cooking techniques (boiling, steaming, stir frying, and microwave) were tested on the changes of targeted phenolic compounds, antioxidant property (ferric reducing-antioxidant power (FRAP) activity), α-glucosidase activity, antinutritive compounds, and sensory properties in commonly consumed traditional leafy vegetables in Southern Africa, the non-heading Chinese cabbage (*Brassica rapa* L. subsp. *chinensis*) and African nightshade (*Solanum retroflexum* Dun). Stir frying increased kaempferol-3-*O*-hydroxyferuloyl-trihexoside, kaempferol-dihexoside, sinapoyl malate, rutin, and isorhamnetin-*O*-dihexoside in Chinese cabbage leaves, followed by steaming. Similarly, stir frying increased kaempferol-3-*O*-rutinoside, chlorogenic acid, caffeoylmalic acid, and quercetin-3-*O*-xylosyl-rutinoside in nightshade, followed by steaming. Biomarkers, sinapoyl malate (Chinese cabbage) and caffeoylmalic acid (nightshade), separated the stir frying from the other cooking techniques. Steaming and stir-frying techniques significantly increased the FRAP activity; whereas boiling and microwaving reduced the tannin, oxalate, and phytate contents in both leafy vegetables and steroidal saponins in nightshade. Stir-fried nightshade leaf extract showed the most effective inhibition against α-glucosidase activity, with an IC_50_ of 26.4 μg ml^−1^, which was higher than acarbose, a synthetic compound (positive control; IC_50_ 69.83 μg ml^−1^). Sensory panelists preferred the stir-fried Chinese cabbage and nightshade leaves, followed by steamed, microwaved, and boiled vegetables.

## Introduction

Consumer preference for the intake of fruit and vegetables in the daily diet is increasing, and the World Health Organization (1) recommends a minimum of 400 g of fruit and vegetables, or five portions, per day excluding the starchy tubers. The United States Department of Agriculture (USDA) (2) guidelines state that an individual must consume one cup (~237 g) of raw or cooked vegetables or two cups of raw leafy greens. These recommendations help in the prevention of non-communicable diseases and micronutrient deficiencies. The number of people affected with type 2 diabetes in Africa was projected to increase to 41.5 million in 2035, and it will be more prevalent in middle aged (40–59) people (3). The crop diversification for sustainable diets, nutrition, and income generation helped to recognize the importance of traditional indigenous vegetables for smallholder crop production and to sustain food and nutrition security (4).

Traditional vegetables, African nightshade (*Solanum retroflexum* Dun) and non-headed Chinese cabbage (*Brassica rapa* L. subsp. *chinensis*), are popularly consumed in the Southern African region. Nightshade (Ca 199 mg 100 g^−1^, Fe 12.8 mg 100 g^−1^) and Chinese cabbage (Ca 27–31 mg 100 g^−1^, Fe 0.5–3.5 mg 100 g^−1^) (5) contain higher levels of Ca and Fe than raw spinach (5, 6). Traditionally, these vegetables are consumed in cooked form. Various cooking methods, such as boiling and steaming, are adopted to improve their palatability and sensory properties (stir frying in hot oil has become a popular cooking method of vegetables due to its convenience, taste preference, and fresh cooking pattern) (7).

Cooking improved the availability of phenolic compounds and antioxidant capacity of vegetables (8). Moreover, the dietary phenolic compounds demonstrated inactivation of carbohydrate digestive enzymes, α-amylase and β-glucosidase, and acted as appropriate anti-hypoglycemic agents (9). Thus, one way to manage type 2 diabetes is *via* encouraging consumption of food rich in anti-hypoglycemic agents. However, types of cooking technique, temperature, and the duration affect the extent of the loss of nutrients (10). Although vegetables are generally cooked in households based on convenience and taste preference, the type of cooking method adopted should be standardized.

In leafy vegetables, tannins, oxalates, and phytates are known as common antinutritive compounds (11). Tannins (polyphenols) form complexes with proteins and make them unavailable for absorption (11). Oxalates bind with dietary calcium and prevent them from being absorbed (11, 12); besides the insoluble calcium, oxalates stored in the kidney manifest as a health-related condition known as “kidney stones.” The negatively charged phosphate groups in phytic acid chelate with Zn or phytates binding with proteins make them unavailable for absorption (11). Different cooking techniques, particularly blanching, reduced the contents of antinutritional factors (11).

Thus, the objective of this study was to investigate the influence of different household cooking methods, such as boiling, steaming, stir frying, and microwaving, on the changes in (i) phenolic components, (ii) antioxidant properties, (iii) antinutritive compounds, (iv) sensory properties, and (v) α-glucosidase activity of Chinese cabbage and nightshade leafy vegetables.

## Materials and Methods

### Materials

Chinese cabbage and nightshade were planted in winter. The leaves (5 kg) were selectively harvested by avoiding leaves that were infected with fungi or infested with pest and washed as described by Managa et al. (13). Thereafter, the leaves were manually chopped into small pieces to mimic the typical domestic preparation and mixed well for homogeneity. Leaf samples (100 g) were selected for four different household techniques as described below. Raw leaf samples (100 g) were freeze-dried (Telstar Lyoquest Freeze Dryer, model 61644 at −55°C) for biochemical analysis.

### Cooking Techniques

The time taken for each household cooking technique was concluded based on interviews and literature-based evidence (14).

#### Boiling

Nightshade and Chinese cabbage leaves (100 g) were boiled in 150 ml of water at 98°C in a covered stainless steel pot on a moderate flame for 15 min, mimicking the traditional method of cooking, drained.

#### Steaming

Nightshade and Chinese cabbage leaves (100 g) were steamed in 250 ml of boiling water in a stainless steel steamer pot (98°C) for 15 min.

#### Microwave Cooking

Nightshade and Chinese cabbage leaves (100 g) of vegetables were placed in a glass dish with 12 ml of water for 15 min in a microwave oven (Defy) (household) working at 2,450 MHz−900 W for 5 min. Afterwards, the vegetables were drained.

#### Stir Frying

10 ml of virgin olive oil was placed onto a preheated pan, and then 100 g of vegetables was placed in the pan and stir fried for 1–2 min. The oil temperature was ranging from 125 to 140°C. The temperature of the vegetables was 100°C after stir frying.

The samples were cooled rapidly on ice-cold water after each of the above-mentioned household cooking technique to stop further post-cooking biochemical changes.

### Chemicals

The analytical standards, chlorogenic acid, catechin, luteolin, epicatechin, and rutin (purity >95%), and other chemicals were purchased from Sigma Aldrich, Johannesburg, South Africa.

### Targeted Phenolic Metabolites

An ultra-high-pressure liquid chromatography (UHPLC) system equipped with quadrupole time-of-flight (QTOF) mass spectrometer (MS) (Waters, Milford, MA, United States) was employed to identify and quantify the predominant polyphenolic metabolites as described by Managa et al. (13) and Ndou et al. (15) without any modifications. Briefly, phenolic compounds were extracted from 50 mg freeze-dried sample of nightshade and Chinese cabbage leaves subjected to different cooking techniques, using 70% aqueous ethanol coupled with ultra-sonication. The identification and quantification of the phenolic components were carried out using a cocktail standard solution comprising chlorogenic acid (y = 1.6315x – 1.8800, *r*^2^ = 0.99, LOQ = 0.52 ppm), catechin (y = 0.0006x + 0.0006, *r*^2^ = 0.99, LOQ = 0.92 ppm), luteolin (y = 0.0005x – 0.0005, *r*^2^ = 0.96, LOQ = 0.45 ppm), epicatechin (y = 0.0008x + 0.0008, r^2^ = 0.99, LOQ = 0.54 ppm), and rutin (y = 0.0007x + 0.0007, *r*^2^ = 0.99, LOQ = 0.42 ppm) due to the unavailability of commercial standards for all the studied compounds. Working range solution from 1 to 1,500 ng ml^−1^ and UV spectra were monitored over a range of 200–400 nm. The cocktail standard solution was prepared in 50% aqueous methanol containing 1% formic acid, and the concentration of phenolic compounds was expressed as mg kg^−1^. Data processing using the TargetLynx software was conducted as described previously by Managa et al. (13) and Ndou et al. (15).

### *In vitro* Antioxidant Activity Using FRAP Assay

The ferric reducing-antioxidant power (FRAP) assay was performed according to the method described by Managa et al. (13) and Mpai et al. (16) without any modifications. Raw and cooked nightshade and Chinese cabbage leaf freeze-dried samples (0.2 g) were extracted with sodium acetate buffer (pH 3.6). The reaction mixture consisted of leaf extract (15 μl) and 220 μl of FRAP reagent solution (10 mmol L^−1^ 2,4,6-tris(2-pyridyl)-1,3,5-triazine (TPTZ) and 20 mmol L^−1^ FeCl_3_). Subsequently, absorbance was read at 593 nm using a microplate reader (CLARIOstar Plus BMG Labtec; Lasec, Cape Town, South Africa). The reducing antioxidant power was estimated using an external standard curve of Trolox and expressed as μmol TEAC 100 g^−1^.

### Antinutritive Compounds

#### Tannin Content

Freeze-dried leaf samples (0.2 g) mixed with 10 ml 1% HCl. The reaction mixture included 100 μl aliquot of the sample extract and 50 μl vanillin–HCl in methanol (5 ml of 8% HCl in methanol and 5 ml of 1% vanillin in methanol) according to the method described by Price et al. (17) and Managa et al. (18) without any modifications. Tannin content was expressed as mg 100 g^−1^.

#### Phytate Content

100 ml of 2.4% HCl was added to the freeze-dried leaf samples (0.5 mg) to extract the phytates. The quantification was performed using Wade reagent (0.03 g monohydrate ferric chloride and 0.3 g sulfosalicylic acid in 100 ml distilled water) as described previously by Latta and Eskin (19) and Managa et al. (18) without any modifications. Phytate content was expressed as percentage.

#### Oxalate Content

Freeze-dried leaf samples (0.1 g) were homogenized with 30 ml 2 mol L^−1^ HCl to extract the insoluble oxalates. Soluble oxalates were extracted with distilled water using leaf samples (0.1 g) according to the standard Association of Official Analytical Chemists (AOAC) method (20) and Managa et al. (18). The CaC_2_O_4_ was precipitated by adding 5% CaCl_2_, and the pellets were collected and washed three times with 0.35 M NH_4_OH and afterwards dissolved in 0.5 M H_2_SO_4_. The resulting solution (combined soluble and insoluble oxalates) was titrated against 0.1 M of KMnO_4_ at 60°C until an extremely faint pale pink color persisted for 15 s. Oxalate concentration was expressed as mg 100 g^−1^.

### Sensory Analysis

Sensory analysis for the Chinese cabbage and nightshade leaves, subjected to different cooking treatments, was performed using a 9-point hedonic scale as described by Managa et al. (18). Color, taste, and aroma were evaluated using an overall acceptance of a 9-point hedonic scale (9 = like extremely, 8 = like very much, 7 = like moderately, 6 = like slightly, 5 = neither like nor dislike, 4 = dislike slightly, 3 = dislike moderately, 2 = dislike very much, 1 = dislike extremely). Untrained sensory panelists (*n* = 16, men and women) within the age range of 20–60, who are familiar with Chinese cabbage and nightshade leaves and consumed these vegetables at least twice a month, voluntarily participated to evaluate the sensory properties of the cooked leaf samples.

### *In vitro* α-Glucosidase Inhibitory Activity

Leaf extract (5 μl) of nightshade and Chinese cabbage leaves prepared at concentrations of 50–250 μg/ml was mixed with 20 μl α-glucosidase solution (50 μg ml^−1^) into a 96-well plate. α-Glucosidase inhibitory effect was measured according to Sagbo et al. (21) without any modifications. Briefly, 60 μl potassium phosphate buffer (pH 6.8; 67 mM) was added to the mixture and incubated at 35°C for 5 min. Subsequently, 10 μl of 10 mM ρ-nitrophenyl-α-d-glucoside solution (PNPGLUC) was added, and the incubation at 35°C was extended for an additional 20 min. Following this, 25 μl of 100 mM Na_2_CO_3_ was added, and the absorbance was read at 405 nm using a microplate reader. The absorbance was measured for both the leaf extracts, acarbose, and the blank control (without α-glucosidase). The enzyme inhibitory activity was expressed as the percentage of α-glucosidase inhibition. The IC_50_ value (i.e., the concentration of nightshade and Chinese cabbage leaf extracts from different household cooking methods that resulted in 50% inhibition of maximal activity) was determined.

### Statistical Analysis

A completely randomized design was adopted with 10 replicates per cooking technique with the experiments repeated twice. One-way analysis of variance (ANOVA) was used to test the significant differences between the different cooking treatments. Means were compared among the treatments by the least significant difference (LSD) test, with *p* < 005, using the Genstat statistical program for Windows 13th Edition (2010) (VSN International Hempstead, UK). The obtained UHPLC-QTOF/MS data were exported for unsupervised principle component analysis (PCA). Following this, supervised orthogonal projections to latent structures discriminant analysis (OPLS-DA) was executed as described by Managa et al. (18).

## Results and Discussion

### Multivariate Analysis and Tentative Identification of Biomarker Metabolites

**Figure 1:**
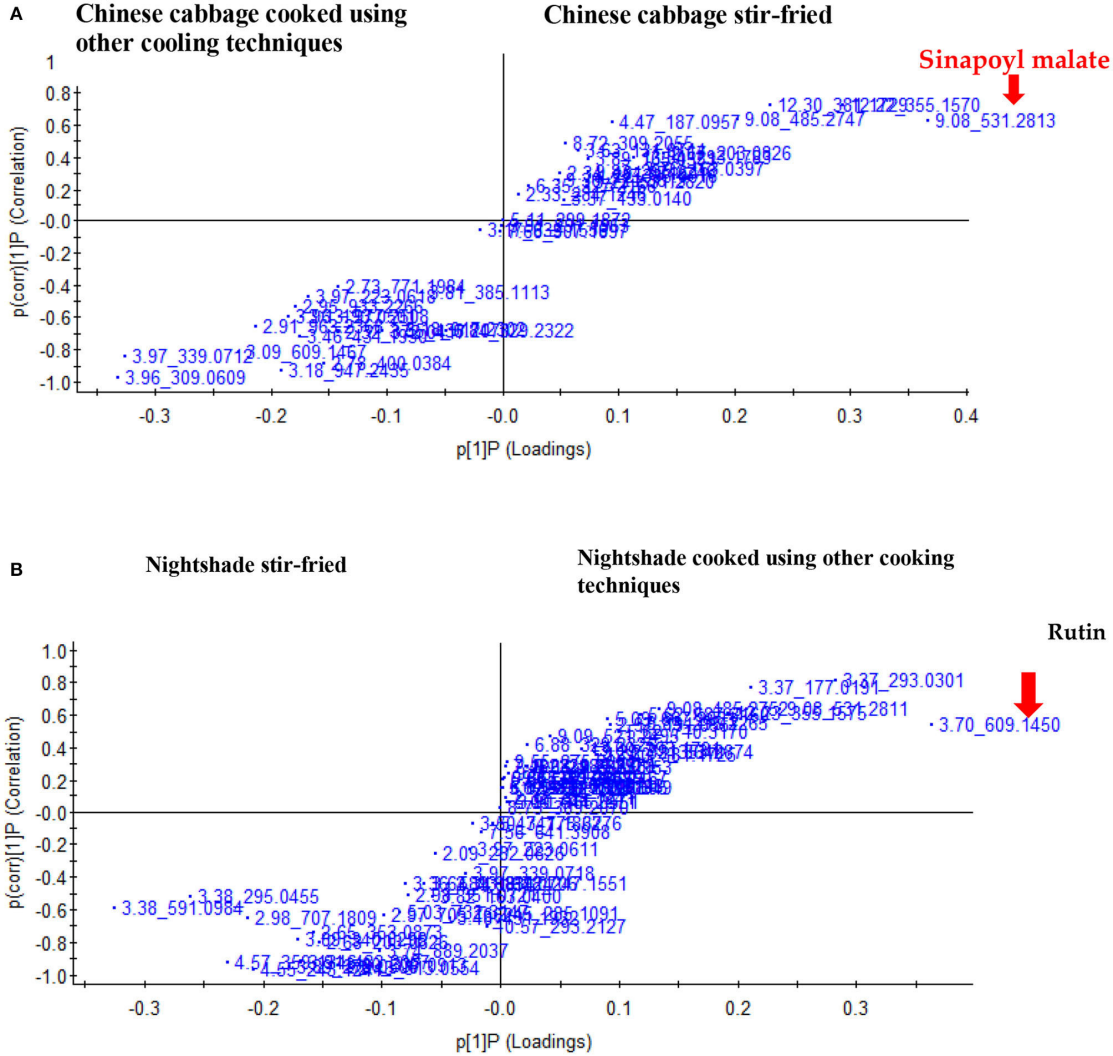
Score plot of orthogonal partial least squares discriminant analysis of UHPLC-QTOF/MS spectra and the marker compounds for the separation of stir-fried leaves of **(A)** Chinese cabbage (*Brassica rapa* L. subsp. *chinensis*) and **(B)** nightshade (*Solanum retroflexum* Dun) from the other household cooking techniques.

Supplementary Figures 1A–C presents the total ion chromatograms scanned in negative ESI-mode for the Chinese cabbage and nightshade leaves subjected to different household cooking techniques. Supplementary Tables 1A,B illustrates the tentative identification of predominant phenolic compounds in both leafy vegetables. Stir-fried Chinese cabbage and nightshade samples contained the highest concentration of total polyphenol content. Chinese cabbage raw leaves contained kaempferol derivatives: kaempferol-sophoroside-hexoside, kaempferol-3-*O*-hydroxyferuloyl-trihexoside, kaempferol-3-*O*-hydroxyferuloyl-diglucoside, kaempferol-dihexoside, kaempferol-3-*O*-sinapoyl-dihexoside-hexoside, isorhamnetin-*O*-dihexoside, isorhamnetin-*O*-hexoside, and sinapoyl malate (Supplementary Table 1A). Raw nightshade leaves contained kaempferol-3-*O*-rutinoside, isorhamnetin-*O*-hexoside, rutin, chlorogenic acid, and its isomer neochlorogenic acid, caffeoylmalic acid, and quercetin-3-*O*-xylosyl-rutinoside (Supplementary Table 1B). The PCA plots indicated two clear groupings, separating the stir frying from the other cooking techniques for both Chinese cabbage and nightshade leaves (Supplementary Figures 1A,B). From the *S*-plots, while sinapoyl malate, a biomarker, separated the stir-fried Chinese cabbage leaves from the other cooking techniques (Figure 1A), rutin separated the stir-fried nightshade leaves from the other cooking techniques (Figure 1B).

### Changes in Phenolic Compounds During Domestic Cooking

The significant influence of four different household cooking techniques on the targeted phenolic compounds in these two vegetables is shown in Tables 1A,B. There was no significant change in the concentration of kaempferol-sophoroside-hexoside between the raw and stir-fried Chinese cabbage leaves (Table 1A). However, a severe decline in the amount of this corresponding compound (~50%) was observed in the microwaved and boiled leaves compared with the raw, stir-fried, and steamed leaves (Table 1A). The concentration of kaempferol-3-*O*-hydroxyferuloyl-trihexoside, kaempferol-dihexoside, isorhamnetin-*O*-dihexoside, isorhamnetin-*O*-hexoside, sinapoyl malate, and rutin was significantly highest (*p* < 0.05) in the stir-fried leaves. Kaempferol-3-*O*-hydroxyferuloyl-diglucoside was significantly (*p* < 0.05) highest in the raw leaves, and a lower concentration was found in the stir-fried leaves. On the contrary, kaempferol-3-*O*-hydroxyferuloyl-diglucoside was completely disappeared in the boiled, microwaved, and steamed leaves (Table 1A). Sinapoyl malate significantly (*p* < 0.05) increased during stir frying, and a relatively low amount was detected after steaming. Sinapoyl malate was not found in the boiled and microwaved leaves.

**Table 1A:** Changes in targeted phenolic compounds in Chinese cabbage (*Brassica rapa* L. subsp. *chinensis*).

**Phenolic compounds (mg kg^**−1**^)**	**Raw**	**Boiling**	**Steaming**	**Microwaving**	**Stir frying**
Kaempferol-sophoroside-hexoside	50.4 ± 0.01^*^^a^	23.77 ± 4.78^c^	42.67 ± 3.03^b^	19.87 ± 3.24^d^	57.98 ± 5.90^a^
Kaempferol-3-*O*-hydroxyferuloyl-trihexoside	4.10 ± 2.77^c^	nd	7.45 ± 4.72^b^	nd	33.44 ± 2.93^a^
Kaempferol-3-*O*-hydroxyferuloyl-diglucoside	27.7 ± 0.05^a^	nd	nd	nd	2.50 ± 0.23^b^
Kaempferol-dihexoside	20.3 ± 0.06^b^	4.84 ± 0.75^d^	16.13 ± 0.25^b^	9.11 ± 4.11^c^	22.69 ± 0.08^a^
Isorhamnetin-*O*-dihexoside	0.13 ± 0.60^d^	0.219 ± 0.0^c^	2.61 ± 0.47^b^	2.41 ± 0.55^b^	6.26 ± 0.0^a^
Isorhamnetin-*O*-hexoside	2.34 ± 0.02^d^	17.39 ± 0.52^b^	33.01 ± 2.59^a^	13.57 ± 5.29^c^	31.65 ± 1.58^a^
Sinapoyl malate	0.04 ± 0.07^c^	nd	21.13 ± 0.10^b^	nd	58.13 ± 48.8^a^
Rutin	7.20 ± 2.77^d^	11.11 ± 5.05^c^	18.68 ± 0.56^b^	18.87 ± 5.75^c^	25.99 ± 3.87^a^
Total polyphenols	112.21	57.329	141.68	63.84	238.64

*Rows with similar alphabetic letter are not significantly different at p < 0.05 according to Fisher's LSD test*.

*nd, not detected*,

* *standard deviation (n = 3, cumulated sample of 10 makes 1 n replicate)*.

**Table 1B:** Changes in targeted phenolic compounds in nightshade (*Solanum retroflexum* Dun) during household cooking.

**Phenolic compounds (mg kg^**−1**^)**	**Raw**	**Boiling**	**Steaming**	**Microwaving**	**Stir frying**
Kaempferol-3-*O*-sinapoyl-dihexoside-hexoside	0.67d ± 0.1^b^	14.72 ± 0.00^b^	nd	11.06 ± 4.59^c^	26.46 ± 3.40^a^
Kaempferol-3-*O*-rutinoside	5.60 ± 0.54^e^	63.97 ± 1.39^d^	81.29 ± 5.50^b^	68.70 ± 6.21^c^	101.18 ± 1.35^a^
Kaempferol-dihexoside	1.63 ± 0.46	nd	nd	nd	nd
Isorhamnetin-*O*-hexoside	0.96 ± 0.23^a^	nd	1.55 ± 0.11^a^	nd	1.29 ± 0.00^a^
Rutin	250 ± 0.23^d^	394.42 ± 1.52^c^	428.04 ± 1.88^ab^	421.68 ± 16.57^b^	440.36 ± 0.12^a^
Neochlorogenic acid	nd	14.72 ± 139^c^	30.23 ± 1.74^b^	10.63 ± 1.04^d^	37.96 ± 2.14^a^
Chlorogenic acid	1.04 ± 0.33^e^	43.03 ± 1.37^c^	86.41 ± 1.09^b^	35.91 ± 3.16^d^	92.60 ± 1.80^a^
Caffeoylmalic acid	3.44 ± 1.81^e^	102.75 ± 1.88^c^	251.97 ± 1.31^b^	87.15 ± 13.75^d^	440.04 ± 1.50^a^
Dicaffeoylquinic acid	35.0 ± 0.21	nd	nd	nd	nd
Quercetin-3-*O*-xylosyl-rutinoside	4.00 ± 1.50^c^	58.28 ± 1.26^b^	76.5 ± 1.70^b^	52.0 ± 0.34^b^	102.46 ± 1.41^a^
Total polyphenols	302.34	691.92	704.03	687.13	1,242.35

*Rows with similar alphabetic letter are not significantly different at p < 0.05 according to Fisher's LSD test. nd, not detected, standard deviation (n = 3, cumulated sample of 10 makes 1 n replicate)*.

Boiled and microwaved methods caused the complete loss of kaempferol-3-*O*-hydroxyferuloyl-trihexoside and kaempferol-3-*O*-hydroxyferuloyl-diglucoside, with the latter significantly (*p* < 0.05) decreased after stir frying compared with the raw leaves. Kaempferol-3-*O*-sinapoyl-dihexoside-hexoside was not detected after the four different adopted cooking techniques, which could be due to the relatively low concentration of this compound in raw material (0.45 mg kg^−1^; Table 1A). Generally, among the studied household cooking techniques, stir frying maintained or even increased the major kaempferol derivatives in Chinese cabbage leaves, probably due to the cooking time related to the different techniques, the stability of kaempferol-3-*O*-hydroxyferuloyl-trihexoside and kaempferol-3-*O*-hydroxyferuloyl-diglucoside was affected. The longer duration of cooking time in this study severely affected the kaempferol derivatives compared with moist cooking (e.g., blanching in hot water at 95°C for 5 min) (22). During domestic food preparation, acylated kaempferol tri-, or tetra-glycosides showed thermal resistance (23). In addition, non-acylated kaempferol diglucosides demonstrated higher loss after boiling and minor loss after steaming broccoli (23), and the degree of loss of these compounds also depends on differences in the texture of different food matrices, such as broccoli florets and Chinese cabbage leaves. The increase of non-acylated kaempferol-dihexoside during stir-frying Chinese cabbage leaves was probably due to transformation of acylated kaempferol-3-*O*-sinapoyl-dihexoside-hexoside through the loss of sinapic acid and further the deglycosylation of a hexoside moiety. It has been reported that acylated kaempferol tri- or tetra-glycosides were more thermally resistant during domestic food preparation (23) than kaempferol-3-*O*-hydroxyferuloyl-trihexoside during domestic cooking. During higher temperature heat treatments, kaempferol-3-*O*-hydroxyferuloyl-diglucoside was expected to degrade to its monoglucoside form; however, either kaempferol-3-*O*-hydroxyferuloyl-monoglucoside or kaempferol aglycone was undetectable. A similar observation was reported during transformation of quercetin-3,4′-*O*-diglucoside in roasted onions (24). The monoglucoside compounds could possibly have been transformed after deglycosylation of kaempferol-3-*O*-hydroxyferuloyl-trihexoside. In addition, the 4′-*O*-glycoside position had a higher thermal stability against deglycosylation than the 3-*O*-glycoside position (24). However, in the present investigation, kaempferol aglycone was not detected at higher temperatures (stir frying). Also, isorhamnetin-*O*-dihexoside could have transformed to isorhamnetin-*O*-hexoside by losing a glucose molecule. Cooking in water (aqueous conditions) at higher temperatures degraded the aglycone compounds into several other simple compounds. In addition, phenolic compounds, such as sinapoyl malate and kaempferol-3-*O*-hydroxyferuloyl-trihexoside, could have leached into the water with higher extents during boiling and microwaving than steaming and stir frying.

In nightshade leaves, kaempferol-3-*O*-rutinoside, kaempferol-3-*O*-sinapoyl-dihexoside-hexoside, rutin, quercetin-3-*O*-xylosyl-rutinoside, and chlorogenic and caffeoylmalic acids increased for all household cooking techniques when compared with the raw leaves (Table 1B). Kaempferol-dihexoside was not detected after cooking. However, kaempferol-3-*O*-rutinoside, quercetin 3-*O*-xylosyl-rutinoside, and chlorogenic and caffeoylmalic acids increased significantly (*p* < 0.05) during stir frying, followed by steaming. Rutin content was detectable at a similar concentration in the steamed and stir-fried nightshade leaves. An increase in rutin and chlorogenic acid contents was also reported during home cooking of sofrito tomato sauce (25). Frying technique at 180°C has been known to increase the availability of chlorogenic acid in Mediterranean vegetables (26). The increase of chlorogenic acid concentration (~88%) observed in the nightshade leaves could be due to the formation of different caffeoylquinic acid isomers during cooking or due to the hydrolysis of dicaffeoylquinic acid (27) as this compound was detected in the raw nightshade leaves (**Table 2B**). A new compound, neochlorogenic acid, that was not present in the raw leaves, was found in all cooking techniques, and its highest concentration was obtained in the stir-fried nightshade leaves. Intramolecular transesterification of 5-*O*-caffeoylquinic acid had produced neochlorogenic acid, a new compound that was not detected in the raw leaves, and it significantly increased after all cooking process and showed the highest concentration in the stir-fried leaves, followed by the steamed leaves. A similarly significant increase in caffeoylquinic acid was reported in fried artichokes compared with the raw and other cooking methods adopted (28). Transesterification of caffeoylquinic acid is dependent on the pH of the food matrix and the temperature and time as well. Neochlorogenic, chlorogenic, and caffeoylmalic acids and rutin were detected after moist cooking (blanching) in our previous investigation and Managa et al. (18); however, the concentrations of the above-mentioned compounds during blanching were much higher than those obtained during domestic cooking (Table 1B). Additionally, the lower pH of the food matrix also exhibited a major role in obtaining higher concentrations of these phenolic compounds after blanching, based on Managa et al. (18). Isorhamnetin-*O*-hexoside was detected in the raw, steamed, and stir-fried leaves without any significant changes (*p* > 0.05) and could possibly have been lost during microwaving and boiling. Additionally, using olive oil for stir frying has been reported to further increase the flavonoids in asparagus spears (29).

### Antioxidant Activity and Different Cooking Techniques

**Figure 2:**
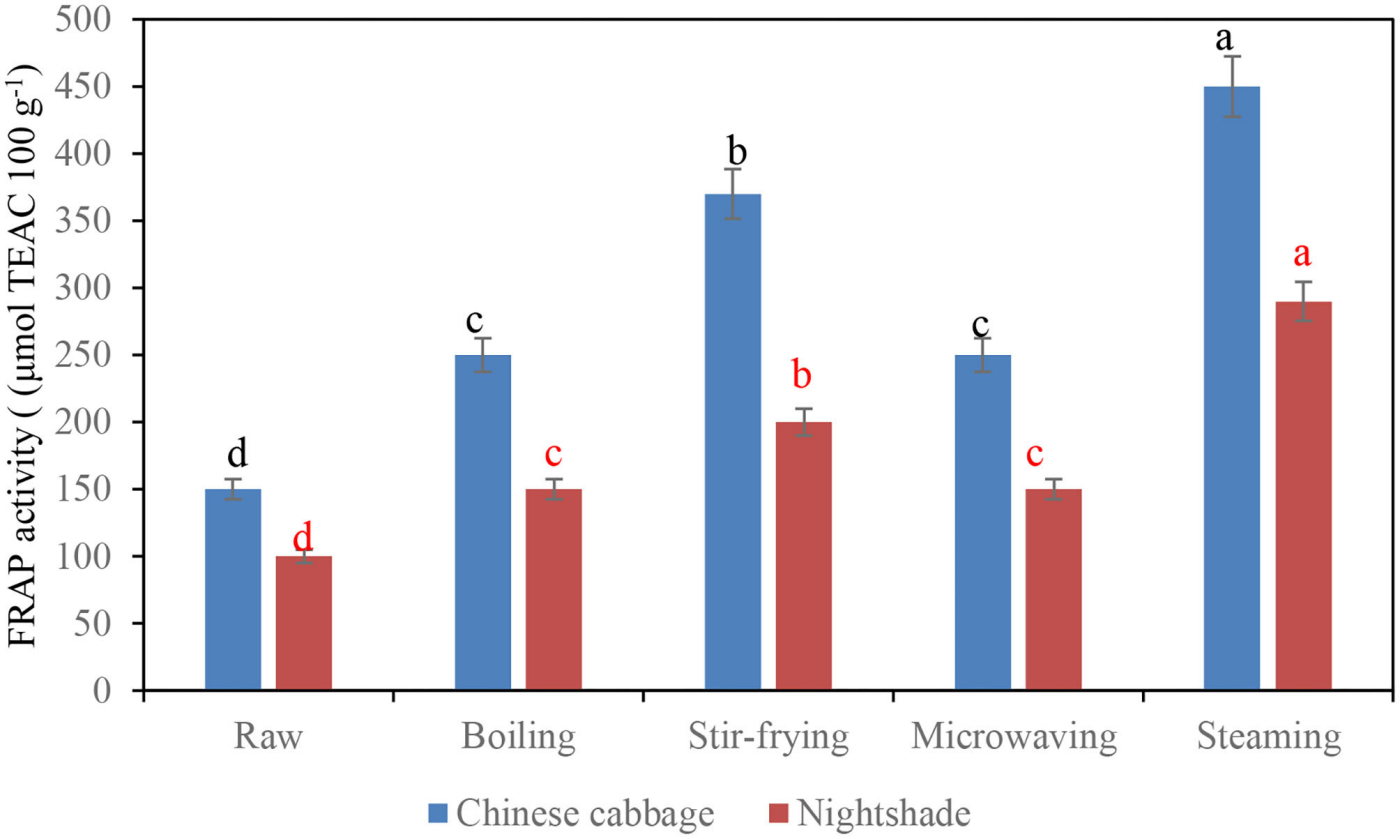
Influence of household techniques on antioxidant property (FRAP) activity of Chinese cabbage and nightshade leaves. Bars with similar alphabetic letter for a particular leafy vegetable are not significantly different at *p* < 0.05 according to Fisher's LSD test.

**Figure 3:**
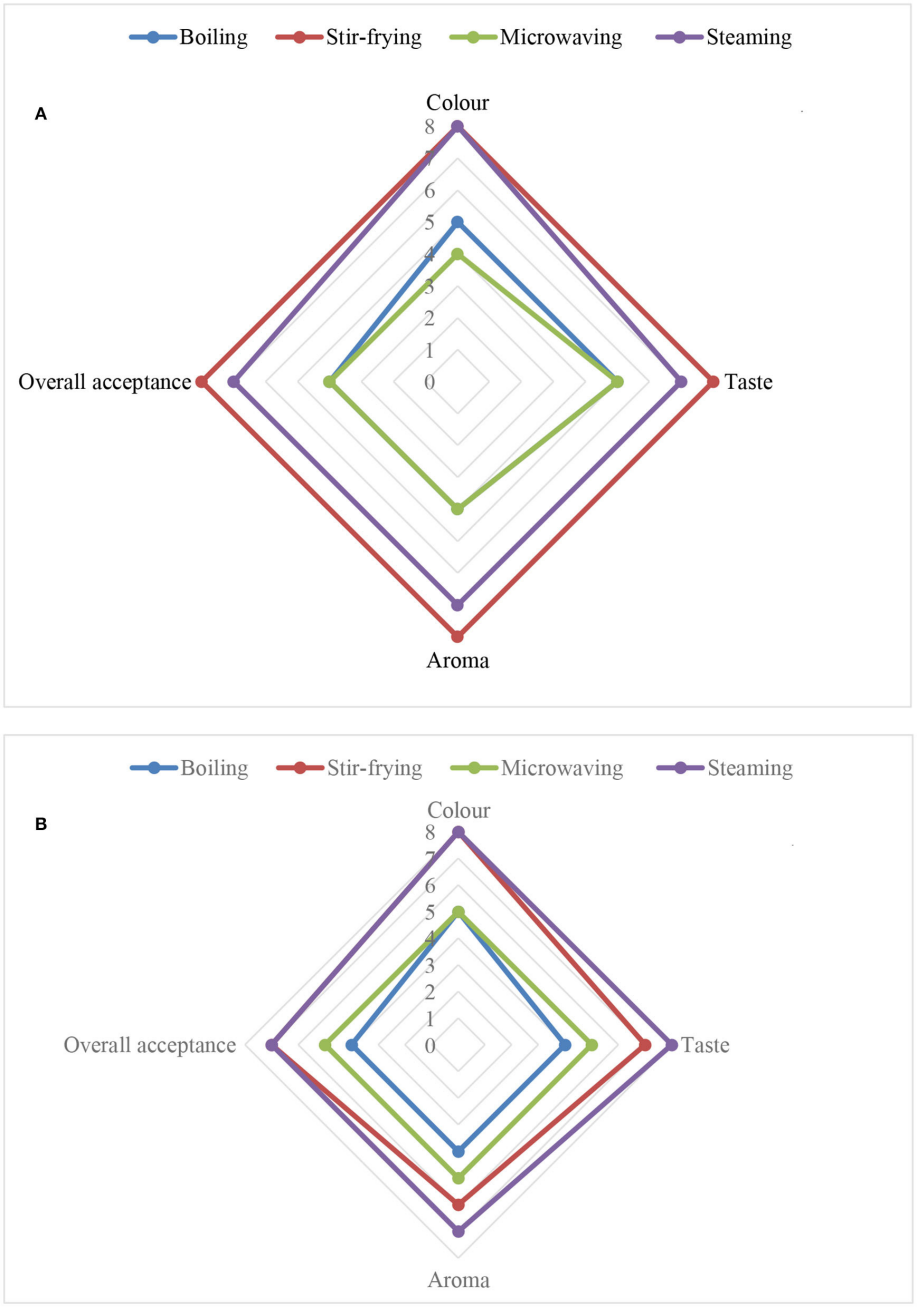
Influence of different household cooking techniques on sensory properties of **(A)** Chinese cabbage and **(B)** nightshade leaves.

The antioxidant property (FRAP activity) of Chinese cabbage and nightshade leaves after different types of domestic cooking techniques is shown in Figures 2A,B. All tested household cooking techniques showed higher antioxidant activity than the raw vegetables. Steaming demonstrated a significant (*p* < 0.05) increase in FRAP activity, followed by stir frying, which could be due to polymerization of phenols during cooking increasing the antioxidant activity. Polymerization of procyanidins was reported to increase the antioxidant activity (28). Furthermore, some other phenolic compounds that could not be identified by the UHPLC-QTOF/MS due to the unavailability of the commercial standards might also be involved and contributed to the increase of FRAP activity. Similarly, steamed artichokes showed the highest antioxidant activity (FRAP) compared with the stir-fried samples (28). Conversely, stir frying increased the FRAP activity in raw Pak Choi, a dark green Chinese cabbage (7). The lowest (*p* < 0.05) FRAP activity was demonstrated in both microwaved Chinese cabbage and nightshade leaves (Figures 3A,B); similarly, Boari et al. (30) revealed lower antioxidant properties of microwaved asparagus. Although individual antioxidants respond differently to the different types of cooking methods, the changes in chemical structure of polyphenols mediated during cooking (thermal treatment) can significantly influence the antioxidant property of traditional vegetables. Glycosylation of flavonoids reduces the antioxidant activity of their corresponding aglycones; however, the effectiveness of the antioxidant property depends on the configuration of hydroxyl groups attached to the flavonoid B and C rings (31). In addition, the number of hydroxyl groups in polyphenol molecular structure determines the effectiveness of the antioxidant property of the molecule (31). During cooking, the release of sugar moieties from the deglycosylation of phenolic triglycosides, resulting in the formation of diglycoside and monoglycoside compounds (due to cellular structural changes), could have contributed toward the antioxidant property (31). Boiling and microwaving in water always induced the highest decrease in phenolic acids and flavonoids, mainly due to greater losses derived from leaching effects (32).

In addition, the interference of tissue matrix could have played a role especially during the extraction of the samples that underwent steaming or stir frying. According to the literature, phenolic compounds and antioxidant activity declined during different cooking treatments with different vegetables (33). However, several studies demonstrated the enhancement of phenolic content and antioxidant activity of different vegetables *via* different cooking techniques (14, 34, 35).

### Sensory Properties and Different Cooking Techniques

Sensory analysis data shown in Figures 3A,B indicated that the panelists “very much liked” the color of the steamed or stir-fried Chinese cabbage or nightshade leaves. They “moderately liked” the steamed Chinese cabbage and nightshade leaves and liked the aroma of the stir-fried Chinese cabbage “very much,” but nightshade leaves “moderately.” The panelists “very much liked” the taste of stir-fried Chinese cabbage and nightshade leaves, but “moderately liked” the steamed leaves. Overall acceptance (liked very much) was highest by the panelists for stir-fried Chinese cabbage leaves, and stir-fried nightshade leaves were accepted moderately, compared with the boiled and steamed samples. The light green color of the boiled and microwaved leaves could be due to the degradation of chlorophyll (18). Panelists' preference was high for stir-fried Chinese cabbage and nightshade leaves probably due to an increase in aroma from the release of volatile compounds associated with cooking temperature. Increased volatile compounds could have been due to the oxidization of fatty acids by lipoxygenase *via* a series of enzyme-like reactions; this requires further investigation. A similar increase in aroma and volatile compounds was reported in Bok Choy (Chinese cabbage, *Brassica chinensis* L; Shanghai Qing) (7).

### Antinutritive Compounds and Different Cooking Techniques

Antinutritive compounds, including tannins, oxalates, and phytates, were found at the highest concentrations in raw (uncooked) nightshade and Chinese cabbage leaves (Tables 2A,B). The concentrations of tannins, oxalates, and phytates decreased with different cooking techniques. Boiling significantly (*p* < 0.05) reduced the tannin, oxalate, and phytate content in Chinese cabbage and nightshade leaves, whereas microwaving significantly (*p* < 0.05) reduced the tannin content in Chinese cabbage.

**Table 2A:** Effect of different cooking treatments on antinutritive compounds in Chinese cabbage leaves.

**Treatments**	**Tannins (mg 100 g^**−1**^)**	**Oxalates (mg 100 g^**−1**^)**	**Phytates (%)**
Raw leaves	57.63 ± 0.66^a^	101 ± 0.58^a^	90.69 ± 2.85^a^
Boiling	29.99 ± 0.17^c^	17.35 ± 0.21^e^	17.02 ± 0.34^e^
Microwave	29.97 ± 0.19^c^	24.2 ± 0.00^d^	23.59 ± 0.39^d^
Stir frying	51.01 ± 0.17^b^	34.1 ± 0.45^c^	31.87 ± 0.69^c^
Steaming	51.24 ± 0.24^b^	44.33 ± 0.09^b^	75.17 ± 0.67^b^

*Means followed by the same letter within the column are not significantly different at p < 0.05 level; standard deviation (n = 3, cumulated sample of 10 makes 1 n replicate)*.

**Table 2B:** Effect of different cooking treatments on antinutritive compounds in nightshade leaves.

**Treatments**	**Tannins (mg 100 g^**−1**^)**	**Oxalates (mg 100 g^**−1**^)**	**Phytates (%)**	**Tigogenin-5G (mg kg^**−1**^)**	**Tigogenin-GG-Rham-Xyl-Xyl (mg kg^**−1**^)**
Raw leaves	55.63 ± 0.38^a^	88 ± 0.00^a^	88 ± 0.00a	nd	0.04 ± 0.00^e^
Boiling	28.58 ± 0.12^e^	55 ± 0.00^d^	22.1 ± 0.06^e^	9.18 ± 1.79^d^	11.89 ± 2.42^c^
Microwave	50.21 ± 0.02^b^	65.95 ± 0.68^c^	67.14 ± 0.11^b^	9.30 ± 0.87^c^	9.52 ± 0.28^d^
Stir frying	37.93 ± 0.71^d^	77.73 ± 0.73^b^	40 ± 0.11^d^	9.78 ± 1.90^b^	19.44 ± 1.79a
Steaming	46.24 ± 0.62^c^	22.23 ± 0.12^e^	44 ± 0.58^c^	10.29 ± 1.46^a^	15.65 ± 0.94^b^

*Means followed by the same letter within the column are not significantly different at p < 0.05 level; standard deviation (n = 3, cumulated sample of 10 makes 1 n replicate)*.

Phytates are thermostable, and the thermal inactivation of phytates takes place at temperatures above 60°C (36); 40% residual activity was reported at 95°C (37). The temperature of four different cooking techniques adopted in this study exceeded 60°C. The decrease in phytate content during cooking could be due to the formation of insoluble complexes between proteins or minerals, and during boiling and microwaving in water, these compounds can leach into the water (38); a similar explanation can be applicable to the loss of tannins.

Oxalate reduction during boiling could be due to its solubility in boiling water, facilitated by the breakage of cells to leak soluble oxalates into the cooking water. Boiling was reported to remarkably lower the soluble oxalate content by 30–87% than steaming (5–53%) in red and green Swiss chard leaves, spinach, and rhubarb stalks (39). Boiling in water also reduced total oxalates in Thai vegetables significantly by 16–79%, also in Mexican vegetables (40) and in chard (*Beta vulgaris*), watercress (*Nasturtium nasturtium-aquaticum*), spinach (*Spinacia oleracea*), and purslane (*Portulaca oleracea* L.) (38). Our data confirmed that boiling was most effective in reducing the soluble oxalate content of Chinese cabbage and nightshade leaves compared with steaming. Therefore, consumption of boiled Chinese cabbage and nightshade leaves may account a lower risk because soluble oxalate levels were markedly reduced during boiled cooking technique. On the other hand, the oxalate content in Chinese cabbage and nightshade leaves was much lower than that in green and red Swiss chad (964–1,167 mg 100 g^−1^ on fresh weight basis) and spinach (1,145 mg 100 g^−1^ on fresh weight basis) (41). It is also important to consider the content of soluble oxalate and the methods used for cooking vegetables when making dietary recommendations for individuals predisposed to kidney stones. Oxalate in foods can affect the bioavailability of minerals, such as Ca. Noonan and Savage (41) had classified foods into three groups based on oxalate:calcium ratio. Vegetables with oxalate:calcium ratio above 2 have high oxalate content and show the ability to bind with Ca from the other foods consumed at the same time. Spinach leaves showed oxalate:calcium ratio >2, indicating higher risk of binding with Ca. Green and red amaranth leaves showed oxalate:calcium ratio <2, suggesting the limited capacity to bind to available Ca from other foods. However, total oxalate/total calcium (mEq) needs to be estimated for Chinese cabbage and nightshade leaves in the future for dietary recommendations.

Steroidal saponins, tigogenin-G-G-G-G-G (5 glucose units attached) and tigogenin-G-G-Rham-Xyl-Xyl (2 glucose + rhamnose + 2 xylose units), were only detected in nightshade leaves (18). The changes in tigogenin-5G and tigogenin-GG-Rham-Xyl-Xyl during cooking are shown in Supplementary Figure 1B and Table 2B. Raw samples contained significantly lower levels of saponins, after which increased significantly during cooking with different techniques. Among the different types of cooking techniques, boiling and steaming demonstrated a reduced degree of accumulation of these two saponins; steaming and stir frying significantly favored the accumulation of these saponins, and steamed leaves contained the highest levels of tigogenin-5G and tigogenin-GG-Rham-Xyl-Xyl. This could be probably due to reduced leaching of steroidal saponins into water compared with boiling and microwaving. Similarly, tigogenin-5G and tigogenin-GG-Rham-Xyl-Xyl were detected in hot water bath after blanching and steaming for 5 min; however, it was not quantified in our previous study (18). The changes in tigogenin-5G and tigogenin-GG-Rham-Xyl-Xyl during cooking are shown in Supplementary Figure 1B and Table 2B. Raw samples contained significantly lower levels of saponins, and it increased significantly during cooking with different techniques. Among the different types of cooking techniques, boiling and microwaving showed a reduced degree of accumulation of tigogenin-5G and tigogenin-GG-Rham-Xyl-Xyl. Steaming and stir frying significantly enhanced the accumulation of these two saponins. Steamed leaves contained the highest levels of tigogenin-5G, whereas stir-fried leaves showed the highest concentration of tigogenin-GG-Rham-Xyl-Xyl. This could be probably due to reduced leaching of steroidal saponins into water compared with boiling and microwaving. Similarly, tigogenin-5G and tigogenin-GG-Rham-Xyl-Xyl were detected in hot water bath blanching and steaming for 5 min; however, it was not quantified in our previous study (18).

Moreover, the solubility of tigogenin-5G and tigogenin-GG-Rham-Xyl-Xyl is affected by temperature and pH (42). It was stated that the solubility of saponins increased in water with temperature, probably due to rupture of cell wall and release of compounds (43). More solubility of saponins has been reported during heating (43). In addition, the tigogenin-GG-Rham-Xyl-Xyl had been extracted more from the stir-fried leaf. However, the longer duration of cooking time can include molecular transformation and could reduce the concentration of tigogenin-5G and tigogenin-GG-Rham-Xyl-Xyl. Leafy vegetables cannot be cooked for a longer duration, such as yams or tubers, and the sensory properties can be affected negatively.

### α-Glucosidase Activity and Different Cooking Techniques

The effectiveness of the inhibitory effect of stir-fried or steamed Chinese cabbage and nightshade leaf extracts on α-glucosidase activity was compared on the basis of their resulting IC_50_ values (Table 3). Stir-fried nightshade leaf extract showed the most effective inhibitory effect on α-glucosidase activity with an IC_50_ of 26.4 μg ml^−1^ (Table 3), whereas stir-fried Chinese cabbage extract with an IC_50_ value of 36.5 mg ml^−1^ showed the second most active of the treatments tested. Steamed Chinese cabbage with an IC_50_ value of 40.6 mg ml^−1^ and nightshade with an IC_50_ value of 38.9 mg ml^−1^ were less active. Acarbose, the positive control used in this study, inhibited the activity of α-glucosidase with an IC_50_ value estimated at 69.83 mg ml^−1^ (Table 3). Our finding is consistent with literature reports, where acarbose was reported to show less inhibition on α-glucosidase activity. Shai et al. (44) and Shettar and Vedamurthy (45) had reported similar observation previously, and Anam et al. (46) had reported the absence of α-glucosidase inhibition by acarbose. Stir-fried nightshade was the most active cooking technique that showed the highest inhibitory effect on α-glucosidase activity.

**Table 3:** Antidiabetic activity of stir-fried or steamed Chinese cabbage and nightshade leaf extracts as determined by α-glucosidase inhibition assay.

**Sample**	**IC_**50**_ α-glucosidase (μg ml^**−1**^)**
Acarbose (positive control)	69.83 ± 0.02^b^
**Chinese cabbage**	
Stir fried	36.50 ± 0.06^b^
Steamed	40.60 ± 0.14^c^
**Nightshade**	
Stir fried	26.38 ± 0.20^b^
Steamed	38.90 ± 0.18^a^

*Each value is expressed as mean ± SD in triplicate experiments. Values are mean ± SD (n = 3). Mean values with different letters are significantly different at p < 0.05 level. The IC_50_ values were measured as the concentration of the test sample required to inhibit the activity by 50% under assayed condition*.

α-Glucosidase is one of the most important carbohydrate digestion enzymes located on the brush-border surface membrane of intestinal cells (46). α-Glucosidase facilitates the production of glucose for intestinal absorption by hydrolyzing the disaccharides and oligosaccharides present in the intestine (lumen) (47). Inhibition of α-glucosidase, a carbohydrate digestive enzyme, is reportedly one of the most important approaches in managing obesity and diabetes (48). Therefore, consumption of steamed or stir-fried Chinese cabbage and nightshade leaves can be beneficial in reducing the risk of type 2 diabetes (48).

The presence of –OH groups in positions 3 (ring C), 7 (ring A), and 4 and 5 (ring B) in the polyphenol molecular structure or C-4 ketonic functional group or C-2–C-3 double bond plays a vital role in the inhibitory effects of the α-glucosidase by binding to the active sites of the enzyme (49). Reportedly, methylation and acetylation of hydroxyl groups reduce the *in vitro* antioxidant and anti-diabetic properties of the flavonoids (50).

### Correlations Between Bioactive Compounds and Associated Functional Properties

Kaempferol-dihexoside, kaempferol-sophoroside-hexoside, chlorogenic, and neochlorogenic acids, and quercetin-3-*O*-xylosyl-rutinoside demonstrated a positively strong correlation with the FRAP activity (Table 4). It is also evident that there is a positive correlation between α-glucosidase activity and bioactive compounds, such as kaempferol-sophoroside-hexoside or kaempferol-dihexoside in the steamed or stir-fried Chinese cabbage leaves (Table 4). Likewise, steamed or stir-fried nightshade leaves also demonstrated a positive correlation between β-glucosidase activity and rutin or chlorogenic acid, caffeoylmalic acid, or quercetin-3-*O*-xylosyl-rutinoside (Table 4). Both steroidal saponins demonstrated a positive correlation with α-glucosidase activity when maltose was used as substrate (Table 4). The hypoglycemic effect of saponins was reported in the root bark of *Berberis vulgaris* Linn (51). Thus, steaming and stir frying improved the inhibitory effect of nightshade and Chinese cabbage leaves against α-glucosidase; it is possible that the synergistic effect of different phenolic compounds and their varying concentrations played a vital role.

**Table 4:** Pearson's correlation coefficients between targeted phenolic components and *in vitro* antioxidant property (FRAP activity) and α-glucosidase activity from stir-fried or steamed Chinese cabbage and nightshade leaves.

**Targeted phenolic components**	**FRAP activity**	**α-Glucosidase activity**
**Chinese cabbage (*****Brassica rapa*** **L. subsp**. ***chinensis*****)**		
Kaempferol-dihexoside	0.75	0.67
Kaempferol-sophoroside-hexoside	0.81	0.74
Isorhamnetin-*O*-dihexoside	0.65	0.62
**Nightshade (*****Solanum retroflexum*** **Dun)**		
Chlorogenic acid		0.92
Neochlorogenic acid		0.80
Quercetin 3-O-xylosyl-rutinoside		0.88

## Conclusions

It is evident from the study that the dietary phenolic compounds, antinutrients, and associated bioactivities of Chinese cabbage and nightshade leaves are significantly altered by different household cooking techniques. The changes in phenolic compounds involved multiple transformation processes including deacylation, deglycosylation, and hydrolysis during household cooking of Chinese cabbage and nightshade leaves. Stir-fried Chinese cabbage and nightshade leaves showed potent antidiabetic activity and can be used as nutraceuticals to control diabetes by preventing the adsorption of glucose in the lumen of the intestine. In addition, further cell toxicity studies must be performed to recommend frequent consumption. Based on our investigation, among the tested cooking techniques, the recommendation is stir frying for traditional African cuisine to maintain optimal health benefits of consumers. This information is valuable for food manufacturers and chefs. However, further studies with more samples from different seasons are recommended to substantiate the results of the present study.

## Data Availability Statement

All datasets generated for this study are included in the article/Supplementary Material.

## Author Contributions

MM performed the experiment, generated the data, and wrote the first draft of this manuscript. AT visualized and validated the data for phenolic compounds and also provided editorial support. JS was responsible for the antidiabetic activity and data. YS was the research collaborator involved in planning and conceptualizing the research. DS was a grant holder and conceptualized the research, supervised the MM, and provided editorial support. All authors contributed to the article and approved the submitted version.

## Conflict of Interest

The authors declare that the research was conducted in the absence of any commercial or financial relationships that could be construed as a potential conflict of interest.
