# Supplementary material for: Impact of Household Cooking Techniques on African Nightshade and Chinese Cabbage on Phenolic Compounds, Antinutrients, in vitro Antioxidant, and β-Glucosidase Activity
Source: Front Nutr. 2020 Dec 21;7:580550. doi: 10.3389/fnut.2020.580550 (PMC7779405; doi:10.3389/fnut.2020.580550)
Supplement: Supplementary file 1 [file Table_15.pdf]

Impact of household cooking techniques on African nightshade and Chinese cabbage on phenolic compounds, antinutrients, in vitro antioxidant and β-glucosidase activity Millicent G.M [1]., Jerry Shai [2]., Anh Dao Thi Phan [3]., Yasmina Sultanbawa[3]., Dharini Sivakumar [1,3]*

[1] Phytochemical Food Network Research Group, Department of Crop Sciences, Tshwane University of Technology, Pretoria West, 0001, South Africa [2] Department of Biomedical Sciences, Tshwane University of Technology, Acadia Campus, Pretoria 0002, South Africa, [3] ARC Industrial Transformation Training Centre for Uniquely Australian Foods, Queensland Alliance for Agriculture and Food Innovation, The University of Queensland, QLD 4108, Queensland, Australia

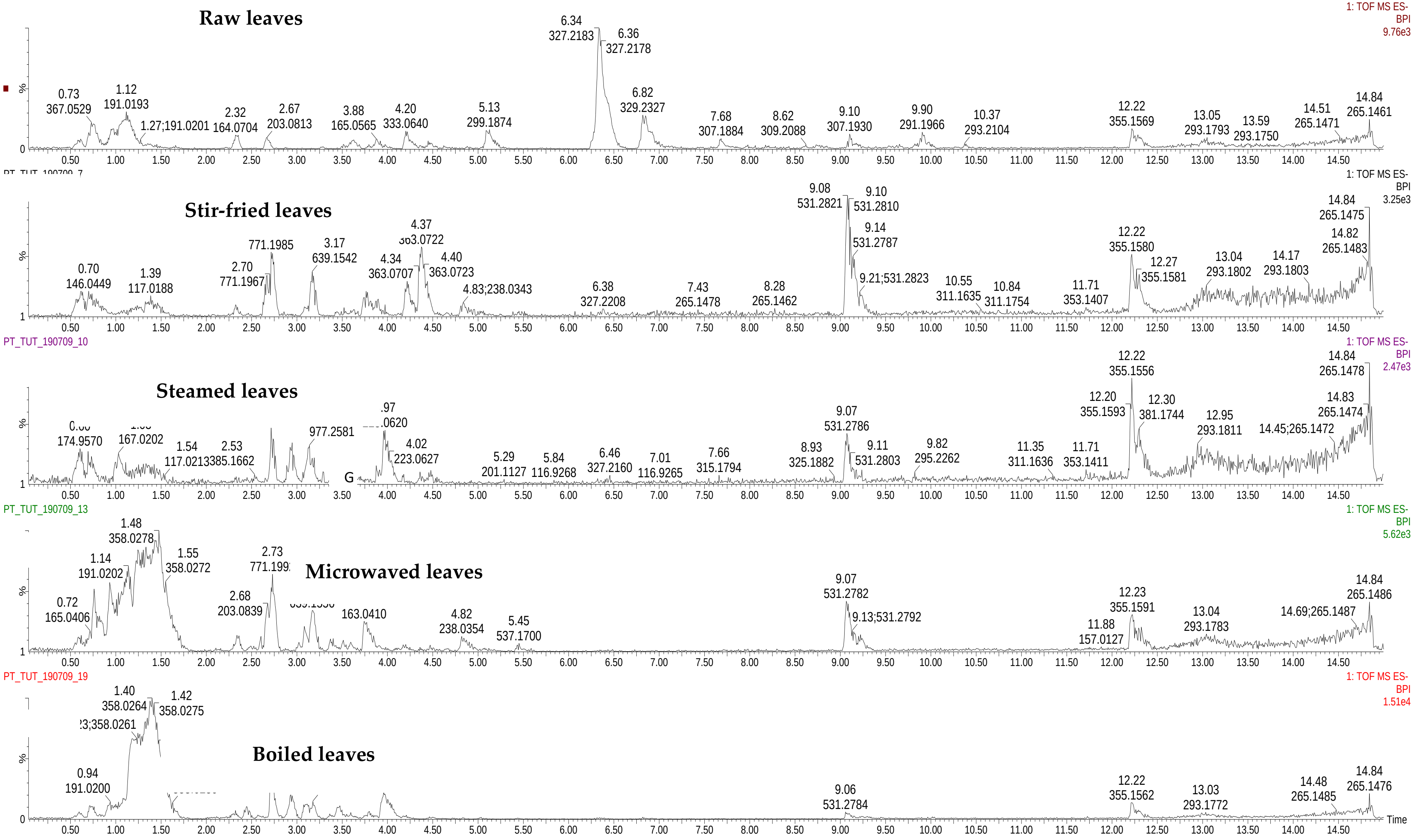

Supplementary Figure 1A: Comparison of UPLC–Q-TOF/MS chromatogram illustrating the changes in phenolic compounds in Chinese cabbage (*Brassica rapa* L. subsp. *chinensis*) during different household cooking techniques. The relative peak intensity is normalized, and peaks are expressed as the percentage highest peak intensity

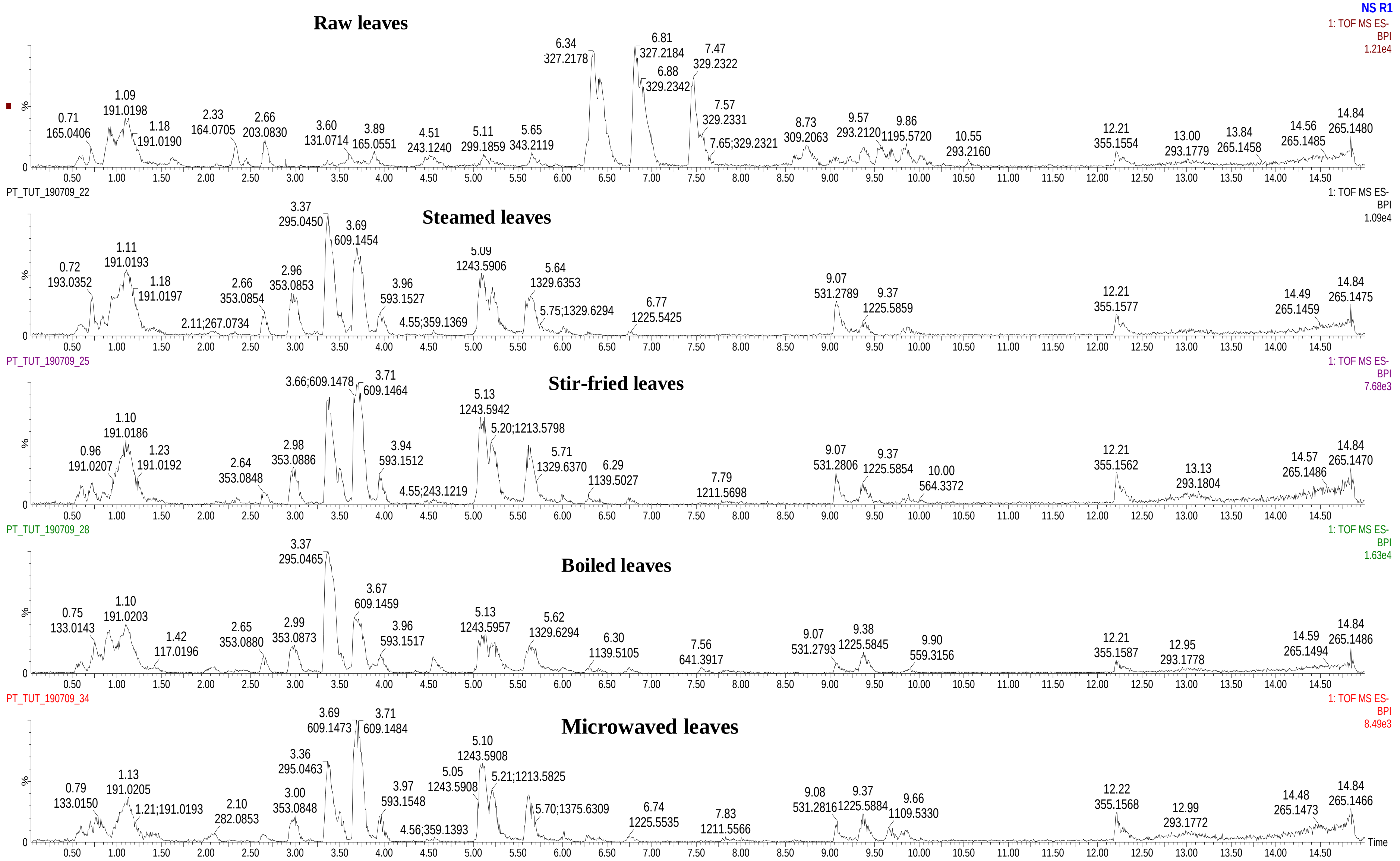

Supplementary Figure 1. B Comparison of UPLC–Q-TOF/MS chromatogram illustrating the changes in phenolic compounds in Nightshade (*Solanum retroflexum* Dun) during different household cooking techniques. The relative peak intensity is normalized, and peaks are expressed as the percentage highest peak intensity.

Supplementary Table 1A. Tentative identification and characterization of phenolic compounds in Chinese cabbage (*Brassica rapa* L. subsp. chinensis) based on their UV (222 to 354) nm retention times relative to the cocktail standards used and the patterns of MS fragmentation under negative mode

Retention time	$[M\text{-}H]^-$	M-H formula	ppm error	MS fragments	UV	Tentative identification	**Ref**
2.71	771.2011	$C_{33}H_{39}O_{21}$	3.5	609,285,255	265,347	Kaempferol 3-O-sophoroside 7-O-glucoside	
2.85	963.2401	$C_{43}H_{47}O_{25}$	-0.5	801,609,285	267,333	Kaempferol-3-O-hydroxy feruloyl-trihexoside	
3.09	609.1474	C_{27}H39O_{16}	3.0	447,285	265,321	Kaempferol-dihexoside	
3.12	977.254	$C_{44}H_{49}O_{25}$	-0.3	815,285	265,333	Kaempferol-3-O-sinapoyl-dihexoside-hexoside	Managa et al., 2019; Managa et al., 2020
3.17	639.1542	$C_{28}H_{31}O_{17}$	-1.6	477,315	254,340	Isorhamnetin-O-dihexoside	
3.45	431.1922	$C_{20}H_{31}O_{10}$	1.2	325,209,207,163,150,133	327	Malic acid derivative	
3.59	801.1874	$C_{37}H_{37}O_{20}$	-0.5	771,609, 285,255	265,340	Kaempferol-3-O-hydroxyferuloyl-diglucoside	
3.96	339.0724	$C_{15}H_{15}O_9$	2.4	309,223,193,164,149,133	265,340	Sinapoyl malate	
4.07	447.1033	$C_{22}H_{21}O_{12}$	0.0	314	253,350	Isorhamnetin-O-hexoside	
4.20	315.05	$C_{16}H_{11}O_7$	-1.6	284,255,151	291	Rhamnetin/Isorhamnetin	
4.63	363.0714	$C_{17}H_{16}O_9$	-0.6	345,285,271	289	Kaempferol-dimethoxy derivative Pubchem 123947790	
6.82	329.2327	$C_{18}H_{33}O_5$	1.2	223,211,171,139,99	275	Unknown	
9.1	531.2821	$C_{26}H_{43}O_{11}$	0,6	485,307	none	Unknown	

7

Table 1B. Tentative identification and characterization of phenolic and steroidal saponin compounds in African nightshade (*Solanum retroflexum* Dun)

Retention time	$[M-H]^-$	M-H formula	ppm error	MS fragments	UV	Tentative identification	References
2.66	353.0851	$C_{16}H_{17}O_9$	-2.5	191,179,135	325	Neochlorogenic acid	
2.98	353.0868	$C_{16}H_{17}O_9$	-2.0	191,85	325	Chlorogenic acid	
3.4	295.0449	$C_{13}H_{11}O_8$	-2.7	179,133	328	Caffeoylmalic acid	
3.73	609.1448	$C_27H_{29}O_{16}$	1.6	300,271	255,352	Rutin	
3.9	593.1519	$C_{28}H_{29}O_{15}$	2.4	285,223,193,149	331	Kaempferol 3-O-rutinoside	
4.12	515.118	$C_{25}H_{23}O_{12}$	-1.9	353,191,179,135	327	Dicaffeoylquinic acid	
4.31	515.1208	$C_{25}H_{23}O_{12}$	3.5	353,191,179,173,135,99	327	Dicaffeoylquinic acid	
5.08	1243.5978	$C_{57}H_{94}O_{28}$	1.9	1081,919,757	none	Tigogenin_5G	
5.16	1213.585	$C_{79}H_{93}O_{18}$	6.8	1081,1051,919,757	none	Tigogenin-3G-Xyl-G	
5.60	1329.6312	$C_{68}H_{97}O_{26}$	-0.7	1081 , 919	none	Tigogenin-GG-Rha-Xyl_Xyl	

6.37	327.2165	$C_{18}H_{31}O_5$	-2.1	229,211,171,97	310	Unknown

A

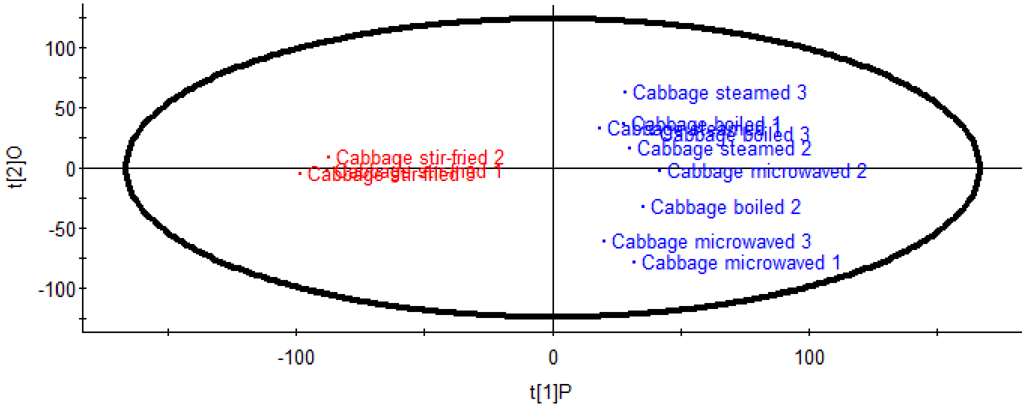

S-DA) - 2020-02-06 09:54:54 (UTC+2)

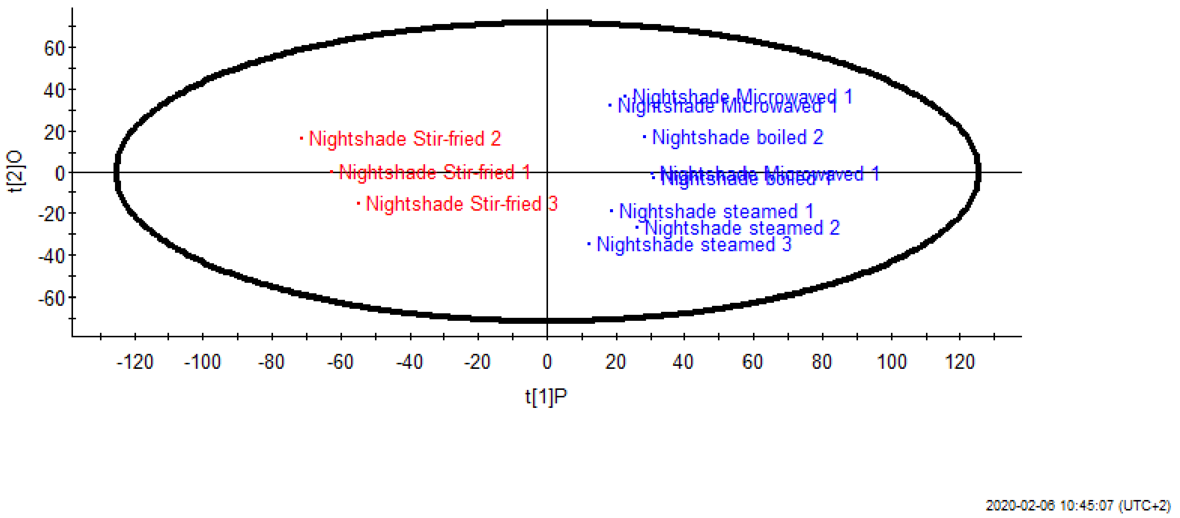

Supplementary Figure. 2: Score plot of Principal component analysis (unsupervised) based on UPLC–Q-TOF/MS spectra of different household cooking techniques A) Chinese cabbage (*Brassica rapa L. subsp. Chinensis*) and B) nightshade (*Solanum retroflexum* Dun)
